# Fluid Restriction for Patients with Heart Failure: Current Evidence and Future Perspectives

**DOI:** 10.3390/jpm14070741

**Published:** 2024-07-11

**Authors:** Naoko P. Kato, Yuji Nagatomo, Fujimi Kawai, Takeshi Kitai, Atsushi Mizuno

**Affiliations:** 1 Department of Health, Medicine and Caring Sciences, Division of Nursing Sciences and Reproductive Health, Linköping University, 581 83 Linköping, Sweden; 2 Department of Cardiology, National Defense Medical College, Tokorozawa 359-8513, Japan; con401@ndmc.ac.jp; 3 Library, Department of Academic Resources, St. Luke’s International University, Tokyo 104-0044, Japan; fjmkw@slcn.ac.jp; 4 Department of Cardiovascular Medicine, National Cerebral and Cardiovascular Centre, Suita 564-8565, Japan; kitait@ncvc.go.jp; 5 Department of Cardiovascular Medicine, St. Luke’s International Hospital, Tokyo 104-8560, Japan; atmizu@luke.ac.jp

**Keywords:** fluid management, fluid restriction, heart failure, review

## Abstract

Fluid restriction has long been believed to benefit patients with heart failure by counteracting the activated renin–angiotensin aldosterone system and sympathetic nervous activity. However, its effectiveness remains controversial. In this paper, we summarized the current recommendations and reviewed the scientific evidence on fluid restriction in the setting of both acute decompensated heart failure and compensated heart failure. While a recent meta-analysis demonstrated the beneficial effects of fluid restriction on both all-cause mortality and hospitalization compared to usual care, several weaknesses were identified in the assessment of the methodological quality of the meta-analysis using AMSTAR 2. Further randomized controlled trials with larger sample sizes are needed to elucidate the benefits of fluid restriction for both clinical outcomes and patient-reported outcomes in patients with heart failure.

## 1. Introduction

Given the aging population and advancements in medical treatment and technology, the number of patients with heart failure (HF) is increasing across the world [1]. HF is not only associated with high mortality rates and frequent hospitalizations; in recent years, it has also been linked to decline in physical ability and frailty. This leads not only to significant healthcare costs but also to increased need for care and burdens on living environments [2]. Recently, novel HF drugs, often referred to as the “fantastic four”, have emerged and are expected to improve the prognosis of HF [3]. However, in the treatment of HF, non-pharmacological treatments, especially self-care, are also considered important and strongly recommended in guidelines in Europe [4], the United States [5], and Japan [6]. Self-care for patients with HF primarily includes adherence to prescribed medications, engagement in physical activity, management of fluid intake and monitoring of symptoms. There is a paucity of research on fluid management in patients with HF. Fluid restriction was traditionally considered a cornerstone of non-pharmacological management in HF patients. Some patients with HF are still advised to limit fluid intake in clinical practice. The rationale for this practice is based on the assumed reduction in body fluids, which could decrease congestion episodes. There is presumption that excessive fluid intake can be a contributing factor to decompensation for HF. However, this recommendation has largely been based on expert opinion. It remains unknown whether the evidence supports fluid restriction in patients with HF.

## 2. The History and Current State of Fluid Management and Sodium Control in Heart Failure

The condition of fluid retention, historically known as “dropsy”, has been recognized since ancient times [7]. Historically, the only treatments available were primitive methods, such as bloodletting and cupping, which physically reduce blood volume [8]. In HF, the main issue is the excessive retention of extracellular fluid, and the advent of diuretics and the recognition of the diuretic effects of digitalis dramatically evolved treatment approaches. Realizing that kidney function plays a crucial role in fluid control constituted a significant advancement.

Since the 1950s, the development of medications with diuretic properties, particularly loop diuretics, has played a pivotal role in fluid management [9]. These drugs, referred to as “diuretics”, primarily function to increase urine output, thereby promoting water excretion. However, it is crucial to note that the excretion of sodium (Na) plays a significant role in their mechanism of action, as it indirectly facilitates the removal of water. Managing sodium is closely linked to fluid management, and dietary sodium restriction has become an important strategy in the treatment and prevention of HF. Modern advancements have made it easier to control dietary salt intake compared to the past, but in clinical practice, it is more convenient to use macro-indicators such as body weight measurements and fluid volume checks.

However, in cases of acute HF, congestion and respiratory distress can occur even without an increase in body weight or a pure increase in body water, and the presence of water retention easily facilitates lung congestion due to increased venous return resulting in augmented cardiac preload. Therefore, managing fluids, weight, and sodium in patients with HF is crucial for preventing its onset and recurrence. In this context, appropriate restrictions on water and sodium intake are recommended. Historically, numerous studies have been conducted on fluid and sodium restriction, not being limited to the use of diuretics. However, loop diuretics, through their actions on the sodium/potassium/2–chloride co-transporter, lead to secretion of renin, with resulting neurohormonal activation [10]. In this context, the opposite concept has emerged and the efficacy of fluid administration in ADHF has also been tested. The evidence on this strategy has been controversial and chaotic. The findings of some small studies have suggested the benefits of co-administration of small volumes of hypertonic saline [11,12]. However, in observational studies, intravenous fluid administration was associated with worse in-hospital outcome [13]. This paper focuses specifically on the clinical safety and efficacy of fluid restriction based on previous studies. We will discuss how fluid restriction is positioned within current treatment strategies and explore the scientific evidence supporting it [14].

## 3. Physiological Changes by Fluid Status in Heart Failure

In the HF setting, the index event on heart function initiates the activation of compensatory mechanisms, including activation of the sympathetic nervous system (SNS) and the renin–angiotensin aldosterone system (RAAS), which are responsible for maintaining cardiac output through increased salt and water retention via reabsorption through the kidneys, peripheral arterial vasoconstriction, increased contractility, and inflammatory mediators that are responsible for mediating cardiac repair and remodeling. In the short term, these systems promote the restoration of cardiovascular function to a normal homeostatic range. However, sustained activation of these systems can lead to secondary end-organ damage in the heart, such as worsened left ventricular (LV) remodeling and subsequent cardiac decompensation. In this context, sodium and water restriction have been believed to be preventive against the promotion of this pathophysiology, since ingested sodium and water are completely absorbed through the intestine [15], irrespective of the presence or absence of HF [16]. The physiological processes outlined above result in the retention of sodium and water in untreated HF patients. This retention serves as the foundation for dietary intake restriction recommendations. Indeed, in untreated pre-clinical HF patients, volume overload by acute saline load showed impaired natriuretic response, which was partially restored by exogeneous natriuretic peptide administration [17]. However, the findings from animal experiments and human studies suggested that fluid restriction [18,19] or sodium restriction [20] can further promote the activation of the RAAS [21] and the SNS. Moreover, these systems mutually activate each other and form a vicious cycle [18,22]. This paradoxical activation of RAAS and SNS activity was also demonstrated in patients with HF [23].

However, in stable HF patients treated with guideline-directed medical therapy (GDMT) such as renin–angiotensin system inhibitors (RASis), beta-blockers, and mineralocorticoid receptor antagonists (MRAs), the renal, hemodynamic, and neuroendocrine responses to alterations in sodium intake did not differ from those observed in healthy individuals [24]. Therefore, it appears that the neuroendocrine mechanism responsible for sensing intravascular volume expansion might be preserved in patients with stable HF patients treated with GDMT. Additionally, strict dietary salt or water restriction is often associated with persistent thirst and reduced food intake [25,26]. In this context, the effects of water restriction might be controversial in the HF setting.

## 4. Current Recommendation on Fluid Intake for Patients with Heart Failure

According to the textbook by Braunwald et al. [14], fluid restriction is generally unnecessary unless the patient is hyponatremic (<130 mEq/L), which may develop because of activation of the renin–angiotensin system, excessive secretion of arginine vasopressin, or loss of salt in excess of water from prior diuretic use. Fluid restriction (<2 L/day) is considered in hyponatremic patients (<130 mEq/L) or for those patients whose fluid retention is difficult to control despite high doses of diuretics and sodium restriction [14]. The descriptions of fluid restriction in HF in the current guidelines are summarized in Table 1. The recent HF guidelines from Japan [6] and the Unites States of America [5] do not address fluid intake recommendations. The guidelines issued by the European Society of Cardiology (ESC) [27] advocate the avoidance of excessive fluid intake in all HF patients. In other words, recent HF guidelines recommend liberal fluid administration for both chronic and acute HF [5,27,28]. Generally, normal fluid intake falls within the range of 1.5–2.5 L/day, corresponding to 15–30 mL/kg/day. In circumstances characterized by hot and humid weather conditions or gastrointestinal fluid loss, it is recommended to increase fluid intake to avoid dehydration [27]. In the case of patients with severe HF or hyponatremia, a fluid restriction of 1–1.5 L/day may be considered to relieve symptoms and congestion [4,5]. Fluid restriction may assist in managing sodium levels in cases of acute decompensated HF (ADHF) patients with dilutional hyponatremia. However, findings from a registry study of acute decompensated HF patients with hyponatremia (the HN Registry) [29] indicate that fluid restriction was the least effective approach for correcting hyponatremia. In Japan, Tolvaptan, a vasopressin type 2 receptor antagonist, has been approved since 2010 for HF patients with volume overload and who are refractory to other conventional diuretic therapies, regardless of the presence of hyponatremia. When employing Tolvaptan treatment for patients with hyponatremia, a liberal fluid intake approach is typically used.

## 5. Challenges and Implications of Fluid Management for Patients with Heart Failure

Previously, it was mentioned that the activation of compensatory mechanisms, including the SNS and the RAAS, alters fluid metabolism in HF patients. However, in stable HF patients treated with GDMT, the neuroendocrine mechanisms for sensing intravascular volume expansion are preserved. Yet in real clinical practice, accurately monitoring intravascular volume is challenging. Various methods, such as chest X-ray, echocardiographic volume measurements, IVC ultrasound, lung ultrasound, and bioimpedance analysis, have been used to assess fluid composition in subjects, including HF patients [30]. Nevertheless, there is no single most-reliable indicator in clinical practice, and self-care monitoring still often relies on body weight as a surrogate marker [31]. In practical recommendations, total fluid volume is often specified for water restriction. However, how these fluids are absorbed and distributed into intravascular and extravascular spaces is unknown. Further research on fluid monitoring using innovative digital devices is anticipated, but for now it remains challenging to regulate appropriate fluid volumes in an aging HF population.

Not only is fluid monitoring difficult, but fluid restriction can also impact a patient’s quality of life. Several studies have shown that fluid restriction is a risk factor for increased thirst distress in patients with HF [32,33]. In addition, it is challenging for HF patients to follow fluid restrictions due to a lack of knowledge about the liquid content of different foods and the management of co-morbidities such as kidney diseases [32,34]. It has been reported that maintaining fluid restriction can be difficult for some HF patients due to their habitual behavior of consuming water with meals. HF patients sometimes have difficulty monitoring daily fluid intake and adjusting fluid intake. Not all patients can adjust their fluid intake based on climatic conditions such as high heat and humidity, or in case of fluid loss, due to nausea and vomiting, for example. Some patients may adhere to fluid restriction even in very hot conditions, leading to dehydration. This is especially common among elderly patients. Moreover, excessive fluid restriction, when combined with diuretic use, can result in dehydration, heightened thirst sensation, and impaired quality of life [33]. Thus, for implementation of fluid restriction, education, support, and planned evaluation are essential. To ensure successful fluid management in patients with HF, it is crucial not only to address the quantity of fluid intake but also to discuss dietary habits, climatic conditions, and diuretic treatment.

## 6. Overview of Randomized Controlled Trials Regarding Fluid Restriction

### 6.1. Studies in Acute Decompensated Heart Failure

In the ADHF setting, the treatment goal is achievement of hemodynamic stability and symptomatic improvement. For this aim, fluid restriction may appear to be a logical intervention. Indeed, it had been traditionally believed to be beneficial and frequently applied to expedite recovery in the management of ADHF [35]. On the other hand, it could be argued that fluid restriction in the setting of intravenous diuretic use could predispose patients to an adverse outcome in certain circumstances, possibly due to the resultant activation of the renin–angiotensin–aldosterone system [36]. Nonetheless, data that could be used to examine this issue are very sparse.

**Figure 1:**
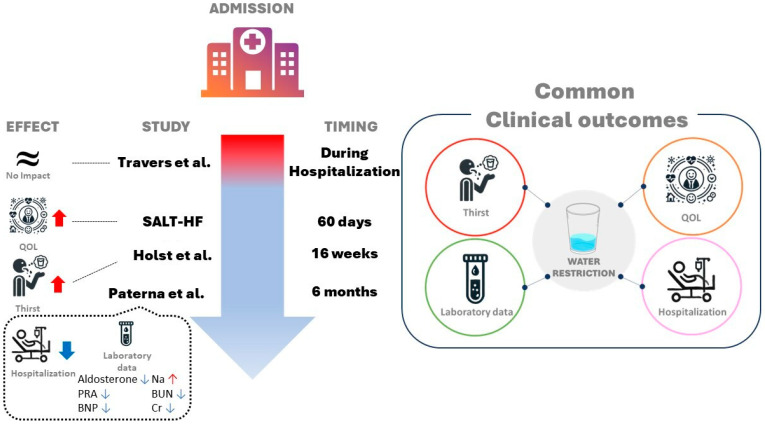
Illustration of the main findings from randomized controlled trials evaluating the effectiveness of fluid restriction in patients with heart failure [38,39,40,41].

Aliti et al. [37] randomized 75 patients to either restricted fluid (<800 mL/day) and sodium (<800 mg/day) intake or liberal fluid and sodium intake (>2.5 L/day and 3–5 g/day, respectively). Weight loss and change in clinical congestion score were similar between both groups. Thirst was significantly worse in the fluid and Na restriction group. Readmission rates at 30 days were also similar. No significant differences were observed between the two groups in terms of intravenous diuretic administration rates, weight changes, or clinical stability during the 3-day follow-up period. Applying a similar approach but concentrating solely on fluid restriction, Travers et al. [38] randomized 67 patients with ADHF within 24 h of admission to either fluid restriction (<1 L/day) or free fluid intake. They observed no significant difference in the time to clinical stabilization between the two groups. Changes from baseline to achievement of clinical stability in serum urea, serum creatinine, B-type natriuretic peptides (BNPs), and sodium did not differ between the two groups. However, the between-group difference in fluid intake was only 400 mL/day (Table 2 and Figure 1).

Albert et al. [39] randomized 46 patients with ADHF and hyponatremia (<135 mmol/L) to 1000 mL/day fluid restriction and usual care at discharge. There were no significant differences in clinical endpoints such as all-cause death, emergency care visits due to HF, or HF rehospitalization. However, the fluid restriction group showed a more favorable quality of life, including symptoms related to HF. Thus, for patients concomitant with hyponatremia, fluid restriction may be beneficial in terms of symptom relief.

Fluid restriction commonly causes thirst in healthy subjects [36] and ADHF patients [37], even in cases of hyponatremia [39], and significantly and negatively impacts quality of life. Xerostomia, altered taste, dry skin, and itching are also seen as side effects in fluid restriction.

### 6.2. Studies in Compensated Heart Failure

There have been only two randomized controlled trials conducted to evaluate the benefits of fluid restriction alone in patients with compensated HF (Table 2 and Figure 1) [40]. Several studies have investigated the impacts of combined dietary interventions involving fluid and sodium restriction [42,43,44,45] or cardiac rehabilitation, emphasizing adherence to diet, physical activity, and fluid restriction [46,47]. The degree of fluid restriction in these interventions varied among studies, with common amounts being 1000 mL and 1500 mL/day.

The study conducted by Holst et al. [40] compared the effects of two different fluid intake regimens: a daily maximum fluid intake of 1.5 L and a liberal fluid intake of 30 mL/kg body weight/day, in patients who had improved from New York Heart Association classification (NYHA) III-IV to stable HF predominantly experiencing mild symptoms (n = 74). This investigation employed a randomized cross-over study design, and the total study duration was 32 weeks, with each intervention period lasting 16 weeks. The study did not provide specific recommendations regarding salt intake. Upon analyzing the end-of-intervention data comparing the prescribed fluid intake of 1.5 L/day and the liberal fluid intake of 30 mL/kg body weight/day, no significant differences were observed in body weight, diuretic usage, other cardiovascular medication, quality of life, physical capacity assessed via the six-minute walk test, or hospitalization rates under the less strict fluid prescription. However, it is noteworthy that sense of thirst [median = 51 vs. 23, *p* < 0.001] and difficulty adhering to the fluid prescription [median = 23 vs. 6, *p* < 0.001] were significantly reduced at the end of the 30 mL/kg/day intervention compared to the end of the 1.5 L/day intervention.

Paterna et al. [41] evaluated the effects of different therapeutic strategies (diuretic doses, sodium diets, and fluid intakes) on hospitalizations after a 6-month follow-up in patients with recently compensated HF who were hospitalized within 30 days. A total of 410 patients with compensated HF (NYHA II) were divided into eight groups according to fluid restriction (1000 or 2000 mL/day), sodium consumption (120 or 80 mmol/day), and furosemide doses (125 or 250 mg twice daily). In their multivariate analysis, a maximum fluid intake of 2000 mL/day was significantly associated with an increased risk of hospital admissions (adjusted odds ratio = 3.82, 95%CI = 2.84–5.14, *p* < 0.01). They also found that a normal-sodium diet (120 mmol sodium/day) with limited fluid intake (1000 mL/day) associated with high doses of loop diuretics (250 mg furosemide bid) could be the most effective treatment compared to other combinations.

## 7. Overview of Systematic Reviews Regarding Fluid Restriction

### 7.1. Systematic Reviews and Meta-Analyses

To explore the influence of fluid restriction on both clinical outcomes and patient-reported outcomes among HF patients, we searched for systematic reviews focused on fluid restriction in HF patients using the following search terms in PubMed in October 2023: “heart failure” [MeSH] AND (“Water restriction” OR “fluid restriction”) AND (“systematic” [Filter] OR “Meta-Analysis” [Publication Type]). These search terms were identified using a priori PICOTS-SD (population, intervention, comparator, outcome, setting, and study design) guidelines to refine the search and decrease noise [48]. The publication period was between 2013 and 2023. As a result, eight systematic reviews were identified. One paper was excluded, as it was written in Spanish. Among the seven remaining systematic reviews and meta-analyses of randomized controlled trials, most focused on dietary interventions, including sodium and fluid restriction [42,43,44,45], or on cardiac rehabilitation, emphasizing adherence to diet, physical activity, and fluid restriction [46]. Only two systematic reviews included analyses to investigate the impacts of fluid restriction alone on clinical outcomes and patient-reported outcomes in HF patients [47,49]. We also checked the Cochrane Database of Systematic Reviews, but we could not find any additional systematic reviews.

In a meta-analysis conducted by Stein et al. [47], 331 HF patients were included from three randomized controlled studies [39,40,41]. They demonstrated that fluid restriction alone significantly reduced the relative risk of both all-cause mortality (relative risk = 0.32, 95% CI = 0.13–0.82, I^2^ = 0%) and hospitalization (relative risk = 0.46, 95% CI = 0.27–0.77, I^2^ = 37%) compared to usual care. Conversely, the combination of sodium and fluid restriction did not exhibit any benefit in terms of mortality (relative risk = 0.92, 95% CI = 0.49–1.73, I^2^ = 7%) or hospitalization (relative risk = 0.94, 95% CI = 0.75–1.19, I^2^ = 0%) compared to usual care. Li et al. [44] also conducted a meta-analysis encompassing five studies, which demonstrated that fluid restriction offers no benefit compared to liberal fluid intake concerning mortality, hospital admission, or thirst in patients with HF. However, it is to be noted that this meta-analysis included two studies in which the intervention comprised both sodium and fluid restriction. Therefore, this study did not clearly evaluate the effects of fluid restriction alone.

Regarding the outcome of “thirst”, two studies utilized a visual analog scale (VAS) [50] to compare a fluid restriction group with usual care [39,40]. In the meta-analysis by Stein et al. [47], the VAS was standardized as a 10-point scale, and they found that fluid restriction significantly increased thirst sensation (weighted mean difference = −2.08, 95% CI = −3.81–0.34, I^2^ = 54%). In contrast, another meta-analysis conducted by Simão did not find significant mean differences in thirst sensation compared with the usual-care group [49]. The mean difference between groups was 9.84 points [95% CI = −27.36–47.04, I^2^ = 90%, *p* < 0.01]. The inconsistencies in the results of these two meta-analyses may be attributed to methodological differences between the studies. However, it is noteworthy that both analyses included the same two studies by Holst et al. [40] and Albert et al. [39]. Holst et al. [40] demonstrated that compensated HF patients (NYHA II) experienced significantly stronger thirst sensation with a 1500 mL/day fluid restriction in a randomized cross-over study (n = 64, median = 51 vs. 23, *p* < 0.001). Conversely, Albert et al. [39] reported no significant differences in the sense of thirst among patients hospitalized for ADHF between a 1000 mL/day fluid restriction group and a usual-care group at 30-day follow-up (median = 50 and 50, *p* = 0.77) nor at 60-day follow-up (40 vs. 50, *p* = 0.60). The inconsistency in the results of these two studies may be attributed to several factors, including variations in the HF status of the participants (compensated or decompensated), differences in study design (such as cross-over design or randomized controlled trial), varying follow-up lengths, and small sample sizes. Therefore, further, larger randomized controlled trials are necessary to comprehensively investigate the impacts of fluid restriction on thirst sensation.

Albert et al. [39] demonstrated that 1000 mL/day fluid restriction resulted in better quality-of-life scores for symptom burden (median = 83.3 vs. 50, *p* = 0.018), overall QoL summary score (median = 72.6 vs. 51.0, *p* = 0.038) and clinical QoL score (median = 75.5 vs. 59.1, *p* = 0.039) at 60 days post-discharge.

### 7.2. Methodological Quality

In recent years, there has been an increasing focus on the significance of quality in systematic reviews. A recent meta-analysis conducted by Stein et al. [47] is, to our knowledge, the first study to have demonstrated the positive impacts of fluid restriction on hospitalization and mortality through meta-analysis. These results could potentially trigger a re-evaluation of current recommendations in HF guidelines. However, there is a risk in uncritically accepting the results of a single systematic review. Therefore, it is essential to first evaluate its quality. The quality of the systematic review was therefore independently assessed by three researchers (AM, YN, and NPK) using the AMSTAR 2 tool [51], which is a commonly used instrument for critically appraising systematic reviews including randomized or non-randomized studies of healthcare interventions. The three researchers concurred that a weakness of this systematic review was the authors’ failure to assess the potential impact of bias risk in individual studies on the results of the meta-analysis. Despite the fact that the majority of studies used for the analysis were flagged as having high levels of overall bias, this aspect remained unaddressed. Another weakness was the review’s failure to assess publication bias, which was attributed to the small number of included studies. Thus, it may be prudent to wait for further evidence before concluding whether fluid restriction reduces the risk of hospitalization and mortality.

## 8. Limitations of Current Evidence

One major limitation of the current evidence on fluid restriction in HF is the scarcity of randomized controlled trials, with studies yielding mixed results. This variability might partly stem from factors such as suboptimal methodological quality, concurrent administration of treatments, small sample sizes, disparate follow-up durations, and climatic differences between regions. Additionally, the current randomized controlled studies were conducted over 10 years ago, primarily in Western countries. Current HF treatments and other differences, including racial and ethnic differences, could influence fluid prescriptions and outcomes. Furthermore, given the aging population, there has been a significant increase in patients with HF with preserved ejection fraction (HFpEF) over the past decade, and these patients often have multiple comorbidities. Aging and multiple comorbidities can influence the effectiveness of fluid restriction, as well as adherence to fluid restriction. Previous randomized controlled trials on fluid restriction for HF primarily focused on patients with HF and reduced ejection fraction (HFrEF), and the effectiveness of fluid restriction for patients with HFpEF remains unclear. Therefore, further studies including patients with preserved ejection are necessary.

In terms of racial and regional disparities, non-Western countries exhibit distinct dietary cultures, climates, and body types. For instance, many Asian countries experience hot climates, and their populations generally have smaller physiques compared to Western individuals. These physical, cultural, and environmental differences could influence the recommended fluid intake and adherence to it. Therefore, studies conducted in non-Western countries are essential for understanding optimal fluid intake and fluid management strategies tailored to these unique demographics.

Moreover, there are still fewer studies assessing patient-reported outcomes. In addition to clinical adverse events, it is crucial to evaluate patient-reported outcomes such as quality of life and thirst sensation. Given the inconsistent results from prior studies regarding the impacts of fluid restriction on quality of life and thirst sensation [39,40], it is essential to assess both outcomes. A scale to assess thirst distress was recently developed [52] which could provide valuable insights into the impacts of fluid restriction in patients with HF.

## 9. The Potential Associations of Novel Therapeutics with Fluid Management in Heart Failure

The advancements over the past decade in pharmacological and non-pharmacological treatments have been remarkable. Not only have novel therapeutics been introduced, but the importance of optimizing HF therapeutics has also been recognized. These treatments may potentially alleviate the need for fluid restriction or impact its effectiveness. These novel therapeutics were shown to have favorable effects on hemodynamics and cardiac function. Further, some of them exert these effects without detrimental effects on RAAS or SNS activity.

Sodium–glucose cotransporter 2 inhibitors (SGLT2is) are shown to improve cardiovascular outcomes [53,54] and are recommended for patients with HF, irrespective of LVEF [55]. SGLT-2is cause diuresis and show a significant synergistic effect on natriuresis under co-administration of loop diuretics [56], although some studies have suggested that the change in plasma volume caused by SGLT-2is might not be sustained in the long term [57,58]. Due to these effects, SGLT-2is can cause significant dehydration [59,60]. As a result, the dose of diuretics was reported to be reduced [61] or subsequent initiation of diuretics occurred less often after SGLT-2i administration [62]. SGLT-2is also showed protective effects on the kidneys, such as inhibition of temporal decline in estimated glomerular filtration rate in patients with chronic kidney disease [63]. Of note, the SNS and the RAAS are not activated or may be inhibited after administration of SGLT-2is [56]. These properties of SGLT-2is may have the potential to alleviate the need for sodium and/or fluid restriction in patients with HF.

Sacubitril/valsartan showed significant improvement of cardiovascular outcomes compared to enalapril in patients with HFrEF [64]. Sacubitril increases natriuretic peptide levels by inhibiting the enzyme neprilysin, which may result in a natriuretic effect [65]. Sacubitril/valsartan significantly reduced the dose of diuretics compared to the enalapril arm in patients with HFrEF in the PARADIGM-HF trial [66]. In the post hoc analysis of the PARADIGM-HF [67] and PARAGON trials [68], renal outcome (>50% decline in estimated glomerular filtration rate or progression to end-stage renal disease) was also improved by sacubitril/valsartan compared to comparators (enalapril or valsartan) [67,68]. These findings suggest that sacubitril/valsartan helps diuresis and exerts renoprotective effects and potentially reduces the need for sodium and fluid restriction in patients with HF.

Vericiguat has emerged as a novel drug for the treatment of HF. Previous studies showed that vericiguat administration did not affect renal function [69], dose of loop diuretics [70], or plasma levels of neurohormonal components, including aldosterone and norepinephrine [71]. Irrespective of its neutral effect on these, vericiguat was shown to cause significant improvement of hemodynamics, including reduced pulmonary artery wedge pressure [72], and was shown to be effective in reducing cardiovascular death or ADHF hospitalization of patients with HFrEF [73]. As a novel non-pharmacological therapy, cardiac contractility modulation (CCM) therapy was found to improve cardiac function without increasing myocardial oxygen consumption through the improvement of calcium handling [74] and facilitate reverse remodeling in patients with HFrEF [75]. Randomized control trials demonstrated that CCM therapy improved the 6 min walk distance, quality of life, and functional status of HF patients who remained symptomatic despite GDMT without an indication for cardiac resynchronization therapy [76]. Although there has been no evidence regarding the effect of these novel therapeutics on fluid management, they may have a potential to favorably affect fluid management in patients with HF.

## 10. Future Directions

Considering the mixed results and the limitations of the systematic reviews, future studies are necessary to determine whether fluid restriction provides beneficial impacts for patients with HF. Several randomized controlled trials such as the FRESH-UP trial (NCT04551729) are either currently underway or planned [77]. The FRESH-UP study is a randomized, controlled, open-label, multicenter trial designed to investigate the effects of a 3-month period of liberal fluid intake versus fluid restriction (1500 mL/day) on the quality of life of outpatients with chronic HF, specifically those classified as NYHA II–III patients. The study aims to randomize 506 patients into two groups of 253 each. The primary outcome is quality of life after three months, assessed using the Overall Summary Score of the Kansas City Cardiomyopathy Questionnaire. Secondary outcomes include measures of thirst distress, clinical summary scores, and safety outcomes such as death and HF hospitalizations. This study will provide crucial insights into the effects of fluid restriction on quality of life and other patient-reported outcomes.

## 11. Conclusions

While fluid restriction has been deemed conceptually important in the management of HF, the evidence supporting its effectiveness is not as robust as that for pharmacological treatments like GDMT, making it challenging to establish recommendations for fluid intake in patients with HF. While a recent meta-analysis demonstrated the beneficial effects of fluid restriction on both all-cause mortality and hospitalization compared to usual care, several weaknesses were identified in the assessment of the methodological quality of the meta-analysis. Further randomized controlled trials with larger sample sizes and consideration of cultural and societal contexts are needed. Additionally, the impacts of fluid restriction should be assessed not only with respect to clinical outcomes, but also with respect to patient-reported outcomes such as thirst and quality of life. Managing fluid intake poses challenges for patients with HF. To ensure successful fluid intake, self-care education addressing both the quantity of fluid intake and adjustment of fluid intake based on self-care monitoring is necessary.

## Figures and Tables

**Table 1:** Current guideline recommendations on fluid intake for patients with heart failure.

Guidelines	Japanese Circulation Society [6]	ACC/AHA/HFSA [5]	ESC [4]
Recommendation	No specific recommendation	Fluid restriction for patients with advanced heart failure and hyponatremia to reduce congestive symptoms	Avoid large volumes of fluid intake Fluid restriction of up to 1.5–2 L/day may be considered in patients with severe heart failure or hyponatremia to relive symptoms and congestion
COR		Class IIb	No COR

ACC, American College of Cardiology; AHA, American Heart Association; HFSA, Heart Failure Society of America; ESC, European Society of Cardiology; COR, class of recommendation.

**Table 2:** Summary of characteristics of randomized controlled studies examining the efficacy of fluid restriction in patients with heart failure.

Author, Year, Country	Design	Sample Size	Patient Population	Age	Female	NYHA (%)	Intervention	Comparator	Follow-Up	Major Findings
Albert et al. [39] (SALT-HF) 2013 USA	Parallel-group, single-blind RCT	46	ADHF, serum sodium ≤137 mg/dL	63	49%	I: 2% II: 13% III: 61% IV: 24%	FR: 1 L/day	Usual care	60 days	QoL (KCCQ) was better in the fluid restriction group No significant difference in morality, readmission rates, emergency care visits, or difficulties adhering to the fluid recommendation
Travers et al. [38] 2007 Ireland	Single-blind RCT	67	ADHF	74 ± 12	31 (46%)	IV: 100%	FR: <1 L/day	Free fluid intake	8 days	No significant difference in time to clinical stabilization, changes in serum urea, creatinine, BNP, or sodium
Holst et al. [40] 2008 Sweden	A randomized cross-over study	74	HFrEF without clinical signs of congestion	70 ± 10	12 (16%)	I: 4% II: 88% III: 8%	FR: 1.5 L/day	30 mL/kg body weight/day	16 weeks	The first sensation (VAS) was stronger and more difficulties adhering to the fluid prescription were observed in the fluid restriction group No significant difference in readmission rates, QoL (MLHFQ, EQ-5D), or 6-MWT
Paterna et al. [41] 2009 Italy	RCT	410	Compensated HF	74–77	258 (63%)	II: 100%	8 groups based on FR (1 or 2 L/day), sodium intake (80 or 120 mmol/day), and furosemide doses (250 mg or 125 mg twice daily)		6 months	Fluid intake of 1 L/day reduced risk of hospitalization

ADHF, acute decompensated heart failure; FR, fluid restriction; HF, heart failure; NYHA, New York Heart Association functional classification; KCCQ, The Kansas City Cardiomyopathy Questionnaire; MLHFQ, The Minnesota Living with Heart Failure Questionnaire; RCT, randomized controlled trial; VAS, visual analogue scale.
